# A psychometric evaluation of the Swedish translation of the Perceived Stress Scale: a Rasch analysis

**DOI:** 10.1186/s12888-023-05162-4

**Published:** 2023-09-22

**Authors:** Alexander Rozental, David Forsström, Magnus Johansson

**Affiliations:** 1 Department of Psychology, Uppsala University, Uppsala, Sweden; 2 Centre for Psychiatry Research, Department of Clinical Neuroscience, Karolinska Institutet, & Stockholm Health Care Services, Region Stockholm, Solna, Sweden; 3 Department of Health, Education and Technology, Luleå University of Technology, Luleå, Sweden; 4 Department of Psychology, Stockholm University, Stockholm, Sweden; 5 RISE Research Institutes of Sweden, Division Safety and Transport, Measurement Science and Technology, Stockholm, Sweden

**Keywords:** Perceived stress scale, Psychometric, Modern test theory, Rasch analysis

## Abstract

**Background:**

Stress reflects physical and psychological reactions to imposing demands and is often measured using self-reports. A widely-used instrument is the Perceived Stress Scale (PSS), intended to capture more general aspects of stress. A Swedish translation of the PSS is available but has not previously been examined using modern test theory approaches. The aim of the current study is to apply Rasch analysis to further the understanding of the PSS’ measurement properties, and, in turn, improve its utility in different settings.

**Methods:**

Data from 793 university students was used to investigate the dimensionality of different version of the PSS (14, 10, and 4 items) as well as potential response patterns among the participants.

**Results:**

The current study demonstrates that the PSS-14 has two separate factors, divided between negatively worded items (perceived stress) and positively worded items (perceived [lack of] control), although with only the negative subscale exhibiting good reliability. Response patterns were analyzed using Differential Item Functioning, which did not find an influence of gender on any of the items, but for age regarding the positive subscale (items 6 and 9). The PSS-10 also demonstrated adequate reliability for the negative subscale, but the PSS-4 was not deemed suitable as a unidimensional scale.

**Conclusions:**

Based on the results, none of the versions of the PSS should be used by sum-scoring all of the items. Only the negative items from the PSS-14 or PSS-10 can be used as unidimensional scales to measure general aspects of stress. As for different response patterns, gender may nevertheless be important to consider, as prior research has found differences on several items. Meanwhile, content validity is discussed, questioning the relevance of anger and being upset when measuring more general aspects of stress. Finally, a table to convert the PSS-7 (i.e., negative items) ordinal sum scores to interval level scores is provided.

**Supplementary Information:**

The online version contains supplementary material available at 10.1186/s12888-023-05162-4.

## Background

Stress is a multifaceted phenomenon referring to the physical and psychological reactions to imposing demands [1]. While there are many definitions of stress in the literature, the concept of stress generally refers to: a) contextual stressors, such as the frequency and impact of stressful life events, b) neuroendocrinological effects, for example the release of adrenal glucocorticoids, and c) cognitive processes, reflecting the subjective perception of stress and subsequent emotional and behavioral responses [2]. In researching stress, measures have relied heavily on the two former perspectives, that is, focusing on either the topography of stressful life events or biological markers. However, given an increased interest in the idiosyncratic appraisal of stressors and how it seems to affect individuals differently, Cohen, Kamarck [3] developed the Perceived Stress Scale (PSS), which intends to assess “the degree to which respondents found their lives unpredictable, uncontrollable, and overloading” (p. 387). This includes queries into more general aspects of stress as experienced during the last month, for example, “…how often have you been upset because of something that happened unexpectedly” (item 1). The original version of the PSS includes 14 items that are scored on a five-point Likert-scale: from 0 (“Never”) to 4 (“Very often”). Items 4–7, 9–10, and 13 are reversed, meaning that they are framed in a positive manner, e.g., “how often have you felt that things were going your way” (item 7). In the original study by Cohen, Kamarck [3] the PSS was administered to two samples of college students (*N* = 332 and 114), and as part of one trial of smoking cessation (*N* = 64). Moreover, additional self-report measures were also used to explore the association between the PSS and other constructs. The results showed that the PSS had good internal consistency, Cronbach’s α = 0.84-0.86 depending on the sample, and moderate to strong positive correlations with symptoms of social anxiety, depression, and physical issues (*r*s = 0.52-0.76). However, the relationship with life events and their impact were lower, *r*s = 0.17-0.49, particularly for the samples of college students. No attempt at studying the dimensionality of the PSS was made.

The PSS has since then been translated to at least 25 languages and been the subject of a large number of psychometric evaluations. Moreover, two shorter versions of the PSS with either 10 (PSS-10) or four (PSS-4) items have also been put forward. The latest systematic review of the field was performed by Lee [4], who found 19 eligible studies, including translations into languages such as Spanish, Turkish, and Arabic, with samples derived from a general population, college students, and patient groups. Overall, the PSS exhibited good internal consistencies, i.e., Cronbach’s α > 0.70, in 11 out of 12 cases for the 14-item version, in all 12 cases for the 10-item version, but only in one out of three cases for the 4-item version. Most investigations of its dimensionality suggested a one-factor solution for the 14-item version, but a two-factor solution for the 10-item version, based on exploratory factor analyses. Confirmatory factor analyses were seldom used, but suggested two-factor solutions for all versions. Lee [4] also argued that the test–retest reliability was satisfactory if the time period between two measurement points was shorter than four weeks, but less satisfactory for longer intervals.

Further research on the PSS has been made during the last decade. This includes several studies of additional samples and translations [5, 6], establishing norms for the PSS-4 [7], using confirmatory factor analysis [8], bifactor analysis [9], and Rasch analysis [10–13]. In line with previous studies, higher scores are repeatedly shown to be associated with negative outcomes, such as increased anxiety, depression, and fatigue [5]. Regarding its different versions and their respective dimensionalities, most research point toward two dimensions. Taylor [8] identified two factors for the PSS-10 in English, referred to as “perceived helplessness” and “perceived self-efficacy” (often called “perceived stress” and “perceived [lack of] control” in the literature), highlighting that the multidimensionality of the PSS can make scores difficult to interpret if stress is in fact conceived as unidimensional and global construct. Likewise, for the Spanish translation, Juárez-García, Merino-Soto [9] argued that the two factors for the PSS-10 and PSS-14 probably are a result of the wording of the items, recommending these to be rephrased into either only negatives or positives, or treated separately. Similar findings have also been put forward by Nielsen, Ornbol [10] regarding the Danish translation, identifying two dimensions on the PSS-10 (i.e., one negative and one positive), albeit still exhibiting problems of model fit. Another study by Nielsen and Dammeyer [11], on a different Danish translation of the PSS-10, confirmed these two dimensions, with improved fit after removing item 6 (“…how often have you felt confident about your ability to handle your personal problems?”). Comparable results have been obtained in other studies of the PSS in English and Spanish [12, 13], suggesting that the 14- and 10-item versions likely consist of two dimensions, with most problems of model fit emanating from the positively worded items (i.e., “perceived [lack of] control”).

### The Swedish translation

Stress is a topic of much debate and research interest, particularly because of its association with many health-related outcomes. Stress has for instance been linked to many somatic conditions and mental distress, such as cardiovascular diseases and depression [2]. Moreover, in many countries, work-related stress has also been gaining more attention. This is particularly true of Sweden where a surge in the incidence of long-term sick-leave caused by exhaustion disorder started two decades ago (a non-traumatic stress-related condition which is included in the Swedish version of the International Classification of Disorders, tenth version) [14]. Measuring stress by means of self-reports has therefore become increasingly important, both in clinical and research settings, with the PSS being widely used for screening purposes and determining the outcome of treatment. For example, in a systematic review and meta-analysis of Internet-based cognitive behavior therapy [15], nine out of 13 identified trials (69.2%) had administered the PSS (three with the 14-item version, five with the 10-item version, and one with the 4-item version).

The first translation and psychometric evaluation of the PSS in Swedish consists of an unpublished report from 1996 by Eskin and Parr [16]. The study does not provide any information about how it was translated from English, but the instrument (14 items) was administered to 87 university students, together with 13 questions about stressful life events that might have occurred during the last six months (e.g., “moving”), and self-report measures of depression and perceived social support. The results indicated that the PSS had good internal consistency, Cronbach’s α = 0.82, no association with stressful life events (*r* = 0.09), a moderate positive correlation with depression (*r* = 0.66), and weak negative correlations with perceived social support that ranged from *r*s = -0.29 for friends, and -0.33 for family. However, no attempt was made to investigate its dimensionality.

Since then, two other psychometric evaluations of the PSS in Swedish have been made. Nordin and Nordin [17] used the same translation as Eskin and Parr [16] but with 10 items, distributing the instrument to 3406 individuals as part of a larger survey study on environmental health issues. The PSS had good internal consistency, Cronbach’s α = 0.80-0.86 (depending on the age range) and revealed moderate to strong positive correlations for depression (*r* = 0.57), anxiety (*r* = 0.68), and exhaustion (*r* = 0.71). Furthermore, an exploratory factor analysis identified two factors, with factor 1 consisting of the negatively worded items (explained variance of 33.8%), and factor 2 being comprised of the positively worded items (explained variance 24.1%). However, Nordin and Nordin [17] argued that due to the lack of a theoretical explanation for a two-factor solution, the PSS should be conceived as a unidimensional and global construct, thus disregarding the obtained factor solution. Meanwhile, Eklund, Bäckström [18] administered the PSS-14 to a small sample recruited via the Internet (*N* = 171) and women with stress-related disorders (*N* = 84), revealing good internal consistencies, Cronbach’s α = 0.90 (Internet-sample) and 0.84 (stress-sample), and moderate negative correlations with mastery (*r* = -0.66), and coping ability (*r* = -0.51). As for its dimensionality, Eklund, Bäckström [18] found a two-factor solution after removing item 12, as determined using a confirmatory factor analysis, but argued that the two factors can be collapsed because of the high correlation between them.

### The current study

In Sweden, the PSS is recommended by many regional authorities responsible for the public healthcare sector in the country, making it popular among clinicians. Yet, there is no consensus on which version to distribute to patients, which may affect how scores are interpreted and compared. Moreover, different response patterns on the PSS might also complicate its use, particularly in relation to gender [11]. For example, administering the Turkish translation of the PSS-10 in a sample of 508 university students revealed that women scored higher than men (Cohen’s *d* = 0.30) [19]. Gitchel, Roessler [20] demonstrated a similar finding, with women scoring higher than men, *d* = 0.24, using an English version of the PSS with 11 items administered among a sample of 1079 adults with multiple sclerosis. More specifically, women scored higher overall and higher on the negatively worded items, but women and men were similar in terms of the positively worded items. In addition, Martinez-Garcia, Nielsen [13], distributing the Spanish PSS-10, found that gender interacted with educational type and year in a sample of 399 university students, such as female students studying for a professional degree on the second year in fact experienced less stress than their male counterparts, regardless of educational type or year. Meanwhile, in a study of the English PSS-14, Ribeiro Santiago, Nielsen [12], Differential Item Functioning (DIF) was explored, i.e., to test the unequal probability of providing a certain response depending on a specific attribute. In relation to gender, this was found for items 1, 3, 6, and 10 (see Table 1 for an overview of the items), which was attributed to gender roles typical of many western countries, such as men being less likely to recognize negative emotions but more likely to demonstrate self-confidence. Similar findings has been put forward by Nielsen and Dammeyer [11] for the Danish PSS-10, illustrating DIF related to gender for item 1 and 3. In other words, gender effects may have to be acknowledged and accounted for when administering the PSS, although it should be noted that other studies have not found such an influence [9, 21–23]. Regarding the Swedish translation of the PSS, no published investigation of different response patterns or DIF exists, which would be helpful given its widespread use among clinicians and researchers with a wide array of patient demographics. It is important to note that differences in response patterns as examined by group differences in sum score levels are not to be taken as evidence for issues with invariance or DIF.

**Table 1:** Items of the perceived stress scale

Item	Swedish	English
q1	Hur ofta har du under den senaste månaden känt dig upprörd på grund av att något oväntat har inträffat?	In the last month, how often have you been upset because of something that happened unexpectedly?
q2	Hur ofta har du under den senaste månaden känt att du inte kunnat kontrollera viktiga saker i ditt liv?	In the last month, how often have you felt that you were unable to control the important things in your life?
q3	Hur ofta har du under den senaste månaden känt dig nervös och stressad?	In the last month, how often have you felt nervous and “stressed”?
q4	Hur ofta har du under den senaste månaden framgångsrikt hanterat vardagsproblem och irritationsmoment?	In the last month, how often have you dealt successfully with day to day problems and annoyances?
q5	Hur ofta har du under den senaste månaden känt att du effektivt kunnat hantera viktiga förändringar som inträffat i ditt liv?	In the last month, how often have you felt that you were effectively coping with important changes that were occurring in your life?
q6	Hur ofta har du under den senaste månaden känt tilltro till din egen förmåga att hantera personliga problem?	In the last month, how often have you felt confident about your ability to handle your personal problems?
q7	Hur ofta har du under den senaste månaden känt att saker och ting gått din väg?	In the last month, how often have you felt that things were going your way?
q8	Hur ofta har du under den senaste månaden tyckt att du inte kunnat klara av allt du skulle ha gjort?	In the last month, how often have you found that you could not cope with all the things that you had to do?
q9	Hur ofta har du under den senaste månaden kunnat kontrollera irritationsmoment i ditt liv?	In the last month, how often have you been able to control irritations in your life?
q10	Hur ofta har du under den senaste månaden känt att du har haft kontroll på saker och ting?	In the last month, how often have you felt that you were on top of things?
q11	Hur ofta har du under den senaste månaden blivit arg på saker som har hänt och som du inte kunnat kontrollera?	In the last month, how often have you been angered because of things that happened that were outside of your control?
q12	Hur ofta har du under den senaste månaden kommit på dig själv med att tänka på saker som du måste göra?	In the last month, how often have you found yourself thinking about things that you have to accomplish?
q13	Hur ofta har du känt under den senaste månaden att du haft kontroll över hur du använder din tid?	In the last month, how often have you been able to control the way you spend your time?
q14	Hur ofta har du under den senaste månaden tyckt att svårigheter har tornat upp sig så mycket att du inte kunnat hantera dem?	In the last month, how often have you felt difficulties were piling up so high that you could not overcome them?

Although previous psychometric evaluations of the PSS in Swedish have contributed to its widespread use, several issues remain to be explored. This relates to its dimensionality and whether a one- or two-factor solution is most appropriate, the reliability of the different versions of the measure, and potential response patterns that may affect its scores. The current study aims to address these issues by applying a modern test theory approach, Rasch analysis, which produces estimates that help to understand measurement validity and reliability from the perspective of both items and persons [24, 25]. For a measure like the PSS this means that the response to a specific item reflects both the individual’s level of stress and the level of stress underlying the item, which is not possible to determine using classical test theory approaches like exploratory factor analysis. Rasch analysis also has the advantages of being more robust against missing data, having the ability to test possible item bias and identify items that do not contribute to the measure, and to investigate potential DIF, such as the unequal probability of providing a certain response depending on a specific attribute like gender [26]. This might further the understanding of the Swedish translation of the PSS and how it can be used, thereby increasing its utility in different settings.

## Methods

### Procedure

The data for the current study was derived from a cross-sectional research project concerning stress and wellbeing of university students in Sweden. It received ethical approval from the Swedish Ethical Review Authority in June 2020 (Dnr: 2020–00555). Participants were recruited via advertisements by three universities in Sweden and posts on various student forums on Facebook, LinkedIn, Accindi, and Instagram. No incentives were provided, e.g., monetary or course credits. A website provided information about ethics, research aims and design, and the procedures surrounding data management. Upon submitting informed consent, participants were forwarded to an anonymous survey managed through Limesurvey. Both the website and the survey itself were available in Swedish and English to reach both Swedish and foreign students, but for the purpose of the current study, only Swedish data on the PSS is used. An overview of the cross-sectional research project can be found in Rozental, Forsström [27], which mainly involved exploring the relationship between different self-report measures on stress and well-being, while the current study only focused on the psychometric properties of the PSS.

### Participants

In total, 793 provided data relevant for the current study, i.e., the PSS, gender, and age, with 533 (67.2%) identifying themselves as female gender, and the average age being 28.8 years (*SD* = 8.2, range 18–65). Additional information about the sample is presented in Rozental et al. (2022).

**Fig. 1:**
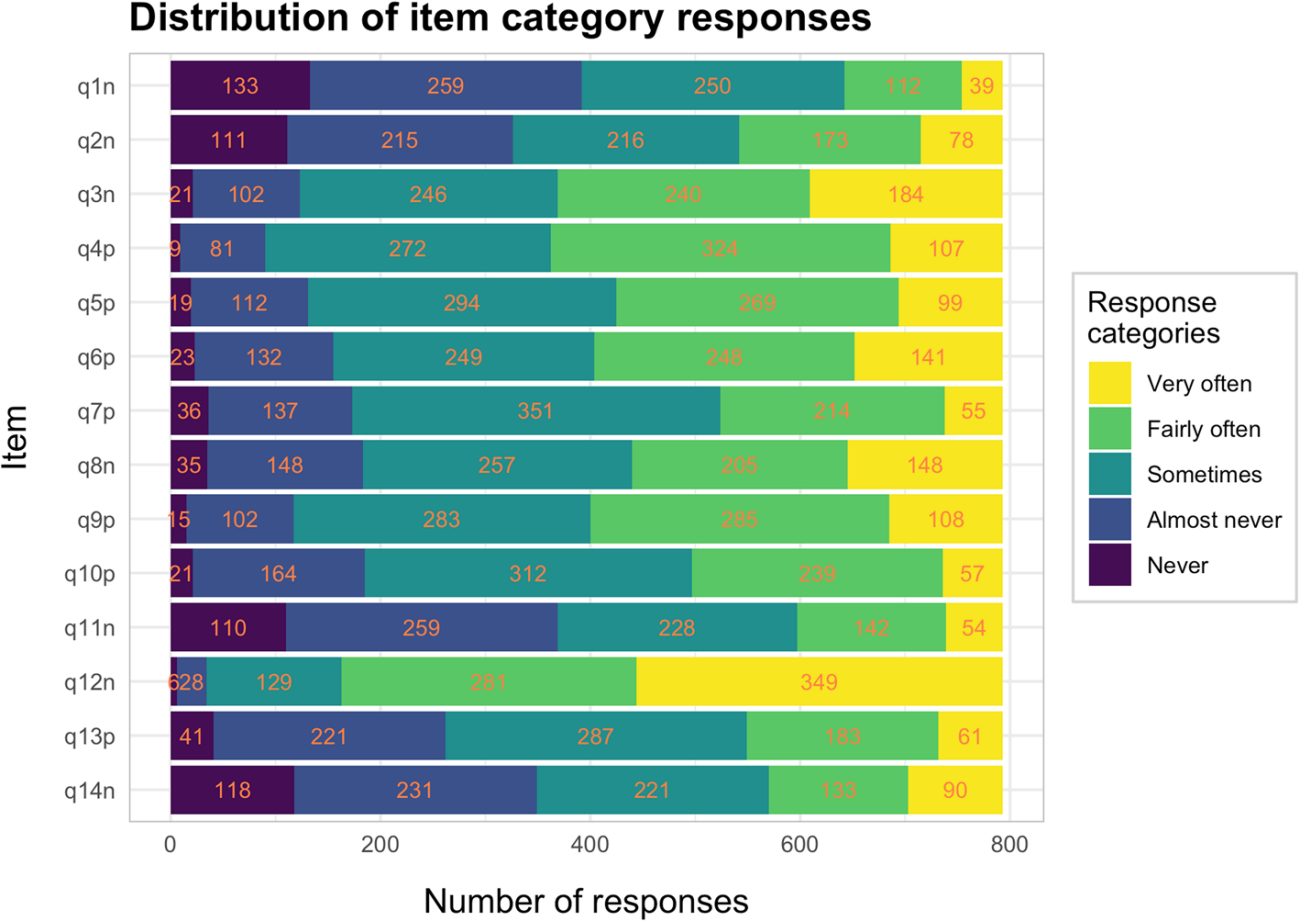
Stacked bar plot of the distribution of response categories for each item

The total number of responses to each response category for all items were 698 (6.3%) for zero, 2191 (19.7%) for one, 3595 (32.4%) for two, 3048 (27.5%) for three, and 1570 (14.1%) for four (see Fig. 1 for item responses).

### Measure

The Swedish translation of the PSS-14 was administered in the current study [3, 16]. It is intended to measure general aspects of stress in various situations, using a time-frame of one month: “…how often have you felt that you were unable to control important things in your life?” (item 2). See Table 1 for an overview of the items in Swedish and English. The PSS utilizes a time-frame of one month and is scored on a five-point Likert-scale, 0 (“Never”), 1 (“Almost never”), 2 (“Sometimes”), 3 (“Fairly often”), and 4 (“Very often”). Additional information about its psychometric properties as found in previous studies can be found in the introduction.

### Analysis

This analysis was structured to evaluate the measurement properties of the PSS using five overarching areas: dimensionality; response categories; invariance; targeting; and reliability [28]. Since the PSS items use multiple response categories, the Rasch partial credit model for polytomous data was used [29]. For an introduction to Rasch analysis methods, we refer to other sources [30–32].

Dimensionality was assessed by multiple methods. A principal component analysis of the Rasch model residuals was conducted, where eigenvalues are expected to be below 2.0 to support unidimensionality [33]. Correlated residuals between item pairs should not be larger than 0.2 above the average of all item-pair residual correlations to ensure that items are independent [34]. Item fit was assessed by unweighted mean square statistics (MSQ) and z-standardized fit statistics (ZSTD), which should be within the range of 0.7–1.3 and ± 2.0, respectively [35]. Since ZSTD is inflated with large samples [36], we used 40 randomly chosen subsamples of *n* = 300 and calculated their average ZSTD values for each item. Item fit also indicates the fit to the Rasch model. Response categories were evaluated by visual inspection of item characteristic curves and item threshold locations to check for disordered thresholds. Targeting of items compared to respondents was reviewed by visualizing the distribution of person locations on the same scale as the distribution of item threshold locations and calculating mean and standard deviations for both distributions. Measurement invariance was assessed by analyzing potential DIF related to gender and age, with 0.5 logits as a DIF size cutoff value [28]. Reliability was assessed by a test information function (TIF) curve and reporting the proportions of respondents located within the scale range where reliability was estimated at or above person separation index 0.7. No point estimate of reliability is reported, since it is more relevant to report the TIF, as it is based on item properties rather than sample properties and describes the variation in reliability across the range of the scale [28].

The Rasch partial credit model with conditional maximum likelihood was used, primarily using the R package eRm version 1.0–2 [37]. DIF analysis was conducted with the R package psychotree version 0.16–0 [38], while response categories and residual correlations were analyzed using the R package mirt version 1.37.1 [39]. Figures and tables were created using the R package RISEkbmRasch version 0.1.16.7 [40]. A fully documented analysis report was made using the scientific publication system Quarto [41]. It includes analysis code and is available on the Open Science Foundation website via the URL provided under Additional Materials. All analyses were conducted using R version 4.2.3 [42] and Rstudio version 2023.06.1 [43].

## Results

### Rating scale

**Fig. 2:**
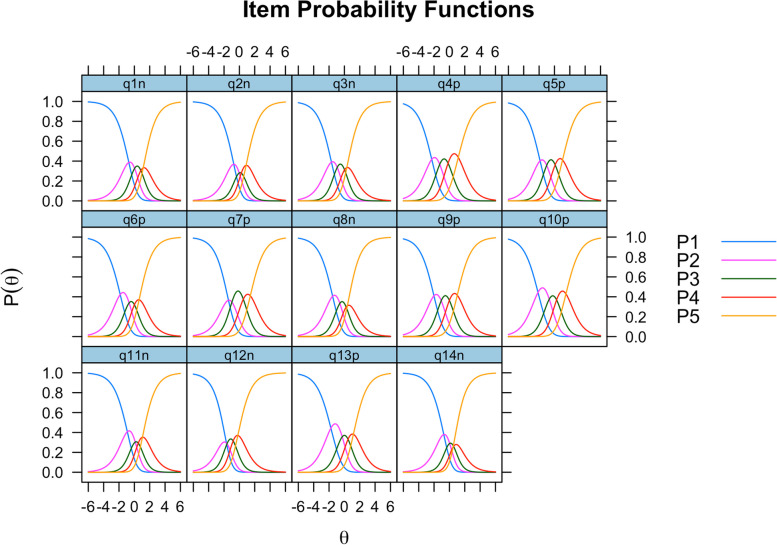
Item probability functions. Note. The y-axis shows probability for each response category, given an individuals’ location on the latent PSS variable shown on the x-axis

Overall, the rating scale adhered to the predefined scale-steps for all of the items, with the exception of item 14 which had minor issues with the second highest category (see Fig. 2). Almost all of the items had notably small distances between item response category thresholds except for items 4, 5, 9 and 10, which showed decent separation of thresholds.

### Differential item functioning

A DIF analysis revealed no issues in relation to gender. Potential differences between those aged ≤ 31 years (*n* = 580) compared to those > 31 years (*n* = 213) were found (*p* = 0.001). Item 6 (“…felt confident about your ability to handle your personal problems?”) and 9 (“…been able to control irritations in your life?”) had DIF size of 0.50 and 0.45, respectively. The younger age group showed lower item locations for both items.

### Scale dimensionality

A PCA of the Rasch model residuals revealed the first and second Eigenvalues at 6.37 and 1.43, which indicates that there are two dimensions in the data. There were also many large residual correlations, which confirms the issues with multidimensionality. See Supplementary File 1 for more information.

**Fig. 3:**
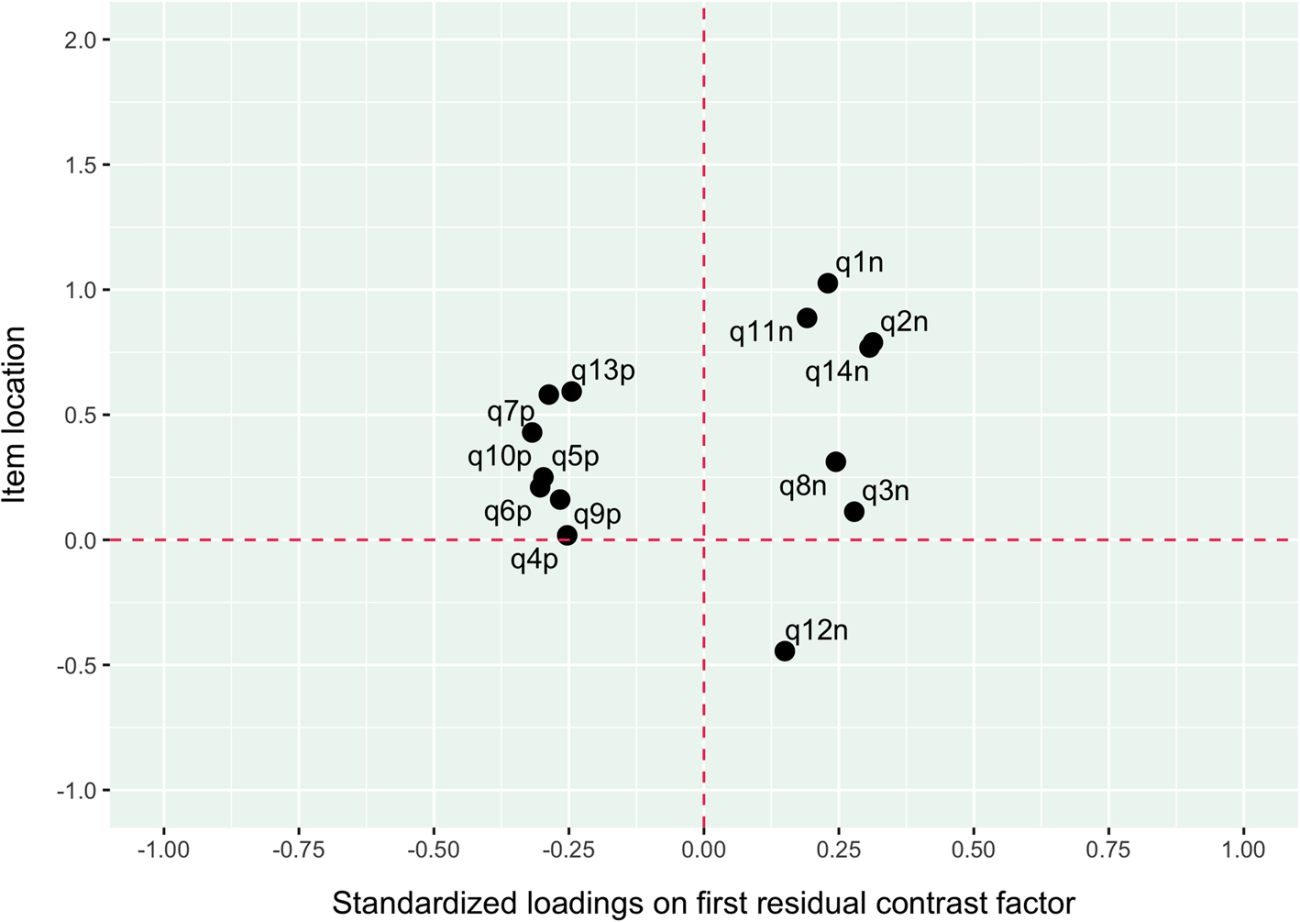
Item locations and loadings on first residual contrast factor

Figure 3 shows the two clusters of items based on their locations and loadings on the first residual contrast factor from the Rasch model. Given the issues regarding multidimensionality, further analyses were made separately for negatively and positively worded items.

### Separate analysis of negatively worded items

The PCA of Rasch model residuals demonstrated lower Eigenvalues compared to the full 14-item measure. No Eigenvalue was above 2.0 (range 0.88–1.77), supporting unidimensionality. Items 1 and 11 were correlated a bit above cutoff value, 0.09. Yet, removing item 11 caused a slightly higher residual correlation between items 1 and 2, which made it reasonable to retain item 11. Item fit was low for items 2 and 3, see Table 2, which did not improve when removing item 11. Notably, the potentially disordered item thresholds for item 14 were not problematic when the negative items were analyzed separately.

**Table 2:** Item fit for the negatively worded items

Item	OutfitMSQ	InfitMSQ	OutfitZSTD	InfitZSTD
q1	0.885	0.898	-1.212	-1.188
q2	**0.638**	**0.634**	**-5.033**	**-5.469**
q3	**0.67**	**0.677**	**-4.693**	**-4.597**
q8	1.002	0.935	0.19	-1.158
q11	1.148	1.152	1.818	1.892
q12	1.128	1.054	0.768	0.791
q14	0.792	0.802	**-2.734**	**-2.83**

**Fig. 4:**
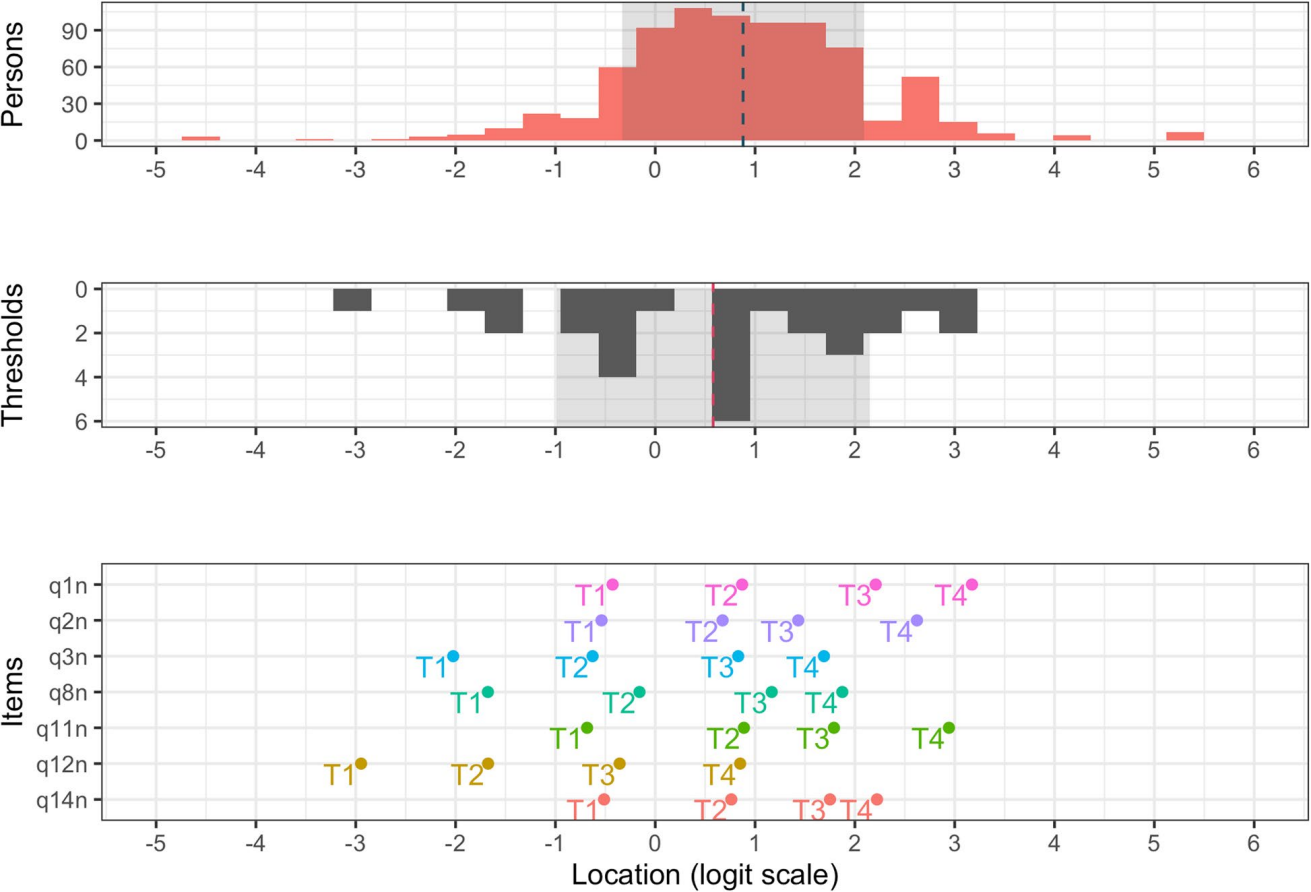
Targeting for the negatively worded items

Overall, most participants are well targeted by items and the sample shows only minor ceiling/floor effects, see Fig. 4. The person/item location descriptives for the seven negative items were as follows: items, *M* = 0.58 (*SD* = 1.57), and persons, *M* = 0.88 (*SD* = 1.57).

**Fig. 5:**
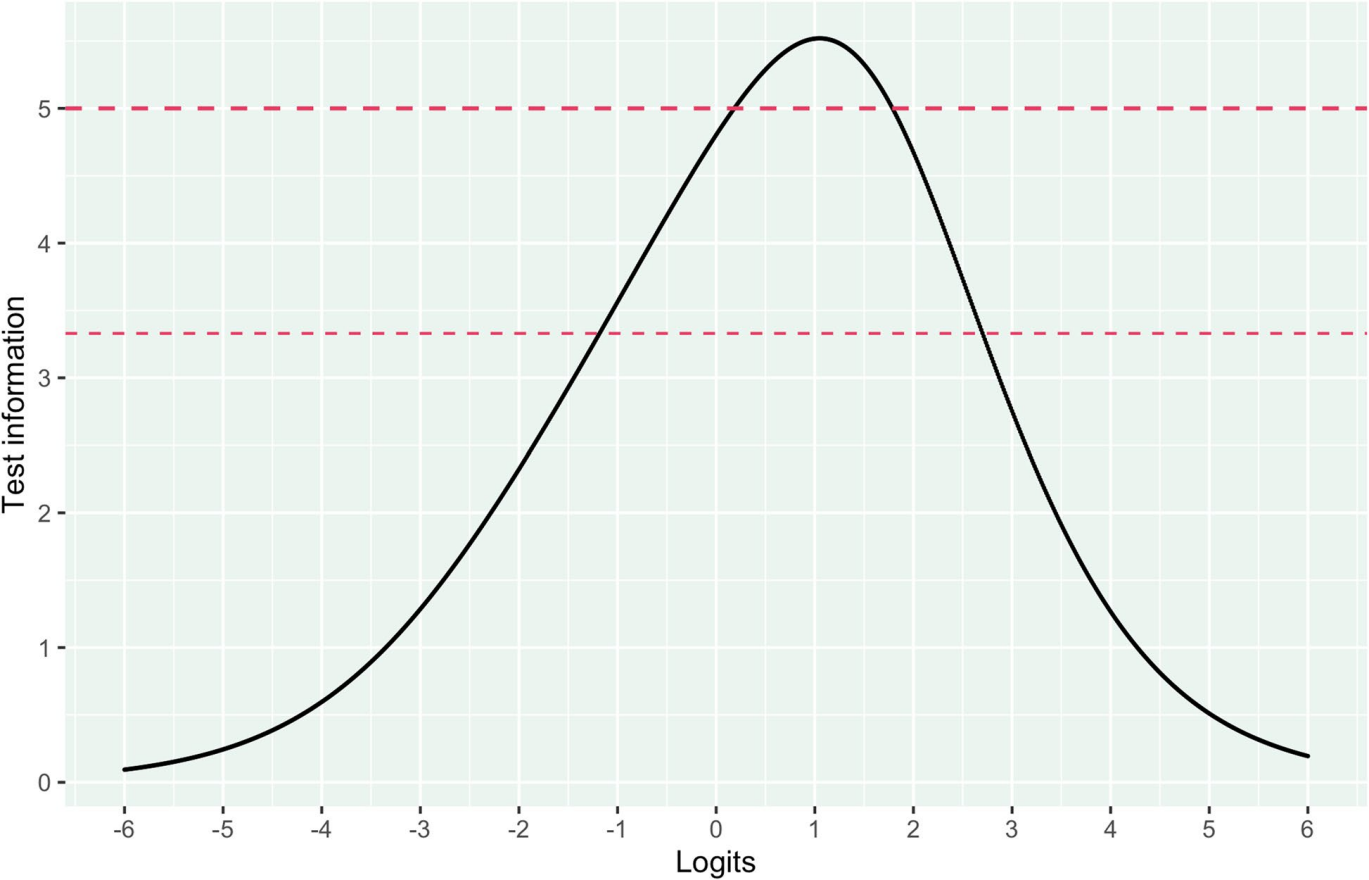
Reliability of negatively worded items. Note. Test information 3.33 and 5.0 corresponds to person separation index of 0.7 and 0.8, respectively

Reliability for the negatively worded items was good. The measure reached reliability (Person Separation Index; PSI) above 0.7 in the interval from -1.19 to 2.7 logits, with 90.1% of the participant locations in this area. 6.2% had person locations above the cutoff for reliability and 2.9% below. Overall, 2.1% were above the highest item threshold (3.17), while 0.5% were below (-2.95), see Fig. 5.

A DIF analysis revealed no issues in relation to gender as no differences of item locations exceeded 0.5 logits. A potential difference between those aged ≤ 20 years (*n* = 59) compared to those > 20 years (*n* = 734) was found for item 12 (*p* < 0.001), but the small sample size obfuscates the results.

**Fig. 6:**
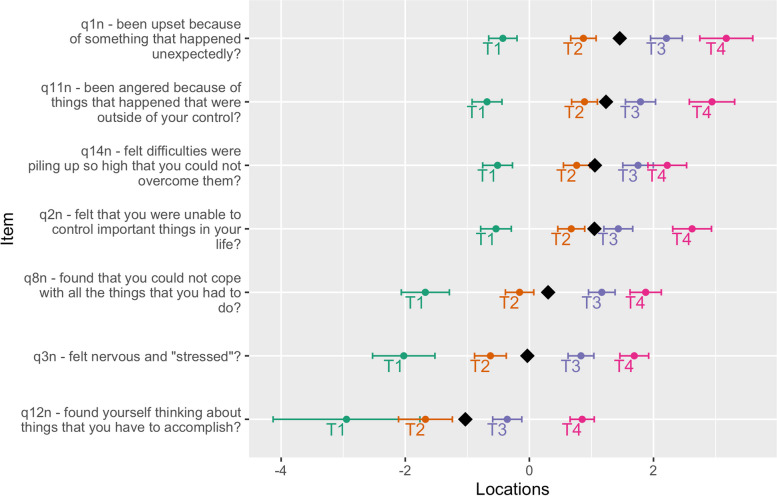
Item hierarchy of the negative items

Figure 6 provides an overview of the item hierarchy for the negative items. Most notably, the two top items (1 and 11) are characterized by content that revolve around anger and being upset, while the rest featured control and coping (items 14, 2, and 8), or being nervous, stressed, and thinking about things that need to be accomplished (items 3 and 12).

See Additional Materials for a link to complete documentation of the analyses made.

### Positively worded items

A PCA demonstrated lower Eigenvalues compared to the full measure, range 0.92–1.69. Item 5 was correlated above cutoff value with both items 4 and 6 (see Table 3), likely due to similar item content, i.e., all involving the ability to successfully deal with life hassles, important changes, and personal problems. Regarding item fit, only item 5 was a bit low (see Table 4). Removing item 5 and running the analysis anew demonstrated no problematic residual correlations, but item fit for item 10 was slightly low.

**Table 3:** Residual correlations for the positively worded items

	**q4**	**q5**	**q6**	**q7**	**q9**	**q10**	**q13**
q4							
q5	0.12						
q6	-0.07	0.12					
q7	-0.19	-0.19	-0.07				
q9	-0.05	-0.17	-0.25	-0.13			
q10	-0.26	-0.21	-0.18	0	-0.13		
q13	-0.27	-0.25	-0.25	-0.24	-0.28	0.03

**Table 4:** Item fit for the positively worded items

	**OutfitMSQ**	**InfitMSQ**	**OutfitZSTD**	**InfitZSTD**
q4	0.882	0.838	-1.542	-1.983
q5	0.669	0.673	-4.55	-4.449
q6	0.708	0.711	-4.015	-4.145
q7	0.861	0.854	-1.811	-1.868
q9	1.032	1.048	0.493	0.531
q10	0.761	0.762	-3.193	-3.162
q13	1.217	1.214	2.488	2.413

After removing item 5, reliability for the positively worded items was poor, barely reaching reliability above 0.7 in the interval from -0.01 to 1.49 logits, with 38.6% of the participants being located in this area. A total of 52.2% had person locations above the cutoff for reliability and 9.2% were below. Overall, 3.8% were above the highest item threshold location (4.16), while 0.5% were below (-2.59).

Since reliability was found lacking and there were DIF issues, no further analyses are reported for the positively worded items. See Additional Materials for a link to complete documentation of the analyses made.

### The PSS-10 and PSS-4

**Fig. 7:**
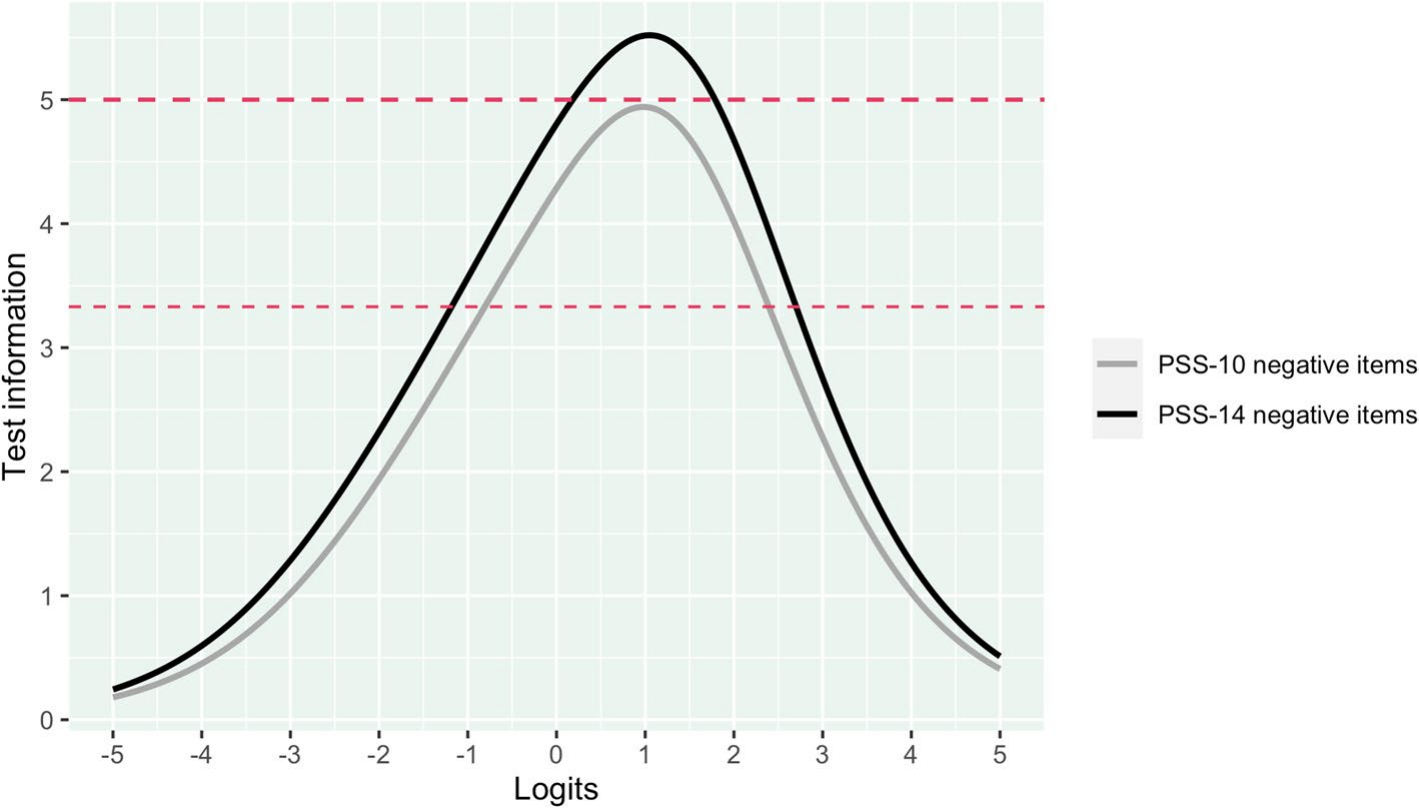
Reliability of negatively worded items for PSS-14 and PSS-10

Separate analyses were made for the shorter versions of the instrument, which are comprised of either 10 and 4 items. For the PSS-10, the results were comparable to the PSS-14, with the same issues related to multidimensionality; i.e., the existence of two dimensions based on negatively and positively worded items. Given the poor reliability and issues with DIF for the positive subscale of the PSS-14, similar findings were also found for the PSS-10 as it consists of the same, but fewer, items (see Fig. 7). Meanwhile, the six items belonging to the negative subscale of the PSS-10 demonstrated decent reliability with 82.1% of respondents located within the scale range where PSI was above 0.7. The negative subscale with six items reached reliability above 0.7 in the interval from -0.804 to 2.388 logits, with 82.1% of the participants being located in this area. Only 7.4% had person locations above the cutoff for reliability and 10.5% were below. Overall, 2.5% were above the highest item threshold (2.98) and 0.9% were below (-2.41). Regarding the PSS-4, analogous issues due to multidimensionality were revealed, which may not be surprising given that it only includes two negatively and two positively worded items. Thus, the PSS-4 is not at all suitable to be used as a unidimensional scale.

To enable estimation of interval level scores based on raw ordinal data, File 2 contains a transformation table to directly convert 7-item PSS ordinal sum scores to interval scores, and File 3 shows item threshold locations, both are available under the Supplementary files.

## Discussion

The current study explored the psychometric properties of the PSS in Swedish by using Rasch analysis. Similar to Nordin and Nordin [17], who employed an exploratory factor analysis for the PSS-10, and Eklund, Bäckström [18] applying a confirmatory factor analysis for the PSS-14, two factors were identified, which are comprised of either negatively or positively worded items. In both of these prior studies the reasoning was nevertheless to regard the instrument as unidimensional, either from a theoretical standpoint or because of the high correlation between the two factors. However, a different perspective is that they actually address different, albeit related, constructs. The negative subscale seems to reflect the concept of negative stress (i.e., distress), e.g., “…felt difficulties were piling up so high that you could not overcome them?” (item 14), in line with the idea of measuring more general aspects of stress [3]. In contrast, the positive subscale seems to convey an ability to effectively deal with stressors, e.g., “…felt that you were on top of things?” (item 10), which instead appears to resemble a sense of mastery. This is in line with prior research [8–13], demonstrating a negative subscale referred to perceived stress and a positive subscale of perceived [lack of] control, which resembles the idea of stress being comprised of appraisal and coping [44]. In line with the recommendations by Juárez-García, Merino-Soto [9], all items should be phrased as either negatives or positives, depending on what concept one intends to study. Researching the negative impact of stress, it seems more reasonable to include only negatively worded items, while the capability to manage stressors is treated as a distinct construct or captured using another self-report measure, e.g., the General Self-Efficacy Scale [45]. Another option would be to treat the two factors separately when administering the PSS, scoring the stress-dimension and the [lack of] control-dimension individually to gain an understanding of both the negative impact stressors might have and the capacity to deal with stressful life events. However, combining the two into a sum score on general aspects of stress is not supported.

As for the two shorter versions, only the reliability for the negative subscale of the PSS-10 was acceptable, analogous to the PSS-14. Hence, should the PSS-10 be used, a similar issue concerning the wording of the items facing the full instrument is important to consider. This is different from the recommendations by Nordin and Nordin [17] who proposed that the PSS-10 could be used in the same manner as the PSS-14 and that it in fact captures a unidimensional and global construct. As for the PSS-4, no previous attempt has been made to determine its reliability in Swedish, but the results from the current study imply that it is unsuitable as a unidimensional scale measuring more general aspects of stress.

In addition to its dimensionality, all items of the PSS adhered to its predefined scale-steps, with the possible exception of item 14 (“…felt difficulties were piling up so high that you could not overcome them?”), having minor issues with the second highest category. In other words, it seems reasonable to retain the present five-point Likert-scale (0–4). However, it should be noted that vague quantifiers, e.g., “Never” and “Often”, are often interpreted differently depending on subjective experiences [46]. This should be particularly true for such a multifaceted phenomenon as stress. Research on how these scale-steps are conceived is therefore recommended, for example by using cognitive interviews [47]. As for response patterns, no difference was observed with regard to gender in the current study. Although similar findings have been obtained in the research [9, 21–23], there are numerous cases were DIF is in fact evident for gender. Ribeiro Santiago, Nielsen [12] found differences in responding to items 1, 3, 6, and 10 in English, and Nielsen and Dammeyer [11] to items 1 and 3 in Danish. This might in turn be attributed to gender roles and their influence on how stress is perceived, experienced, and responded to, which should be accounted for when administering the PSS. The reason as to why similar findings were not demonstrated in the current study is unclear. Data in the current study was collected from university students who may differ from population-wide samples, warranting further research on the subject of gender differences and stress in Sweden. The current study did on the other hand find an influence of age. This was true for item 12 (“…found yourself thinking about things that you have to accomplish?”), comparing those under or above the age of 20, although this needs to be interpreted cautiously given the small sample size. More crucial is perhaps the age effect found for all positively worded items (with the exception of item 6), and particularly item 4 and 9. It might be that individuals over the age of 31 share experiences that are different from those who are younger based on their life situation, such as being more likely to have children and struggling with work-life-balance. Those over the age of 43 may in turn experience their circumstances differently from those being 32–43 years. Further research on response patterns and age should be conducted as it might reflect differences in perceived difficulties of the items. This could also account for such factors as occupation, marital status, and social support. Juárez-García, Merino-Soto [9] for example found a relationship between both age and occupation and negatively worded items, suggesting that younger people as well as workers experience more stress than older people and university students.

Finally, the issue of content validity is relevant to consider. As demonstrated by the item hierarchy for the negative items (see Fig. 6), the two items at the top featured aspects of anger and being upset. Irritation, frustration, and anger are often considered signs of a non-traumatic stress-related condition, including exhaustion disorder [14]. However, these are not symptomatic for everyone experiencing stress, may be more evident in the initial phase of exhaustion disorder, and seem only weakly associated with the underlying construct of exhaustion disorder in the Karolinska Exhaustion Disorder Scale [48]. In a recent study on the topic, responses to open-ended questions about symptoms of exhaustion disorder were completed by 670 participants and analyzed using qualitative content analysis also suggests that frustration/irritability may not be the most typical signs of the condition, as experienced by individuals themselves. Instead, other mood and emotional symptoms were more common, such as depression, emotional regulation, anxiety, and worry [49]. Hence, the PSS might benefit from replacing the two items on anger and being upset, should it be used to measure more general aspects of stress.

There are some limitations important to considering when reviewing the results of the current study. First, while a sample size of 793 is deemed sufficient to conduct Rasch analysis, the constitution of the participants is restricted in that it was based on data from university students. Although being relatively heterogenous in gender and age, those studying in higher education may differ from a working population or specific patient groups when it comes to experiencing and dealing with stressful life events. Previous research on the PSS have used similar samples (c.f., [9, 19]), but the results from the current study should nevertheless be replicated in other settings. Second, given that the participants were self-recruited, this might have created self-selection bias. Because recruitment was made via the Internet and being completely anonymous, investigating the motifs to participate is not possible, including factors that may have affected their responses to the PSS, e.g., motivation, stress levels, or other present living conditions. However, it is plausible that the purpose of the cross-sectional research project, i.e., stress and wellbeing of university students in Sweden, might have attracted individuals that are more interested in the topic, or alternatively suffer from difficulties related to stress and wellbeing.

## Conclusion

The current study examined the Swedish translation of the PSS, demonstrating that items were divided along the lines of being negatively or positively worded. If the instrument is to be used to measure more general aspects of stress, negatively worded items should be treated separately to reflect the unidimensional underlying construct, as compared to the positively worded items which instead seem to capture the concept of perceived [lack of] control. DIF for age was found for the positively worded items, possibly reflecting differences in life situation. However, this was not demonstrated for gender, although previous research has found DIF for several items and might influence the utility of the PSS. was. Taken together with the low reliability, the positively worded items are not recommended for use as a separate unidimensional scale. Finally, the reliability of the six negative items from the PSS-10 is similar to PSS-14 for the negative items subscale, making both useful as brief unidimensional scales without the positively worded items. The PSS-4 is however not recommended due to issues with multidimensionality and low reliability.

### Supplementary Information

**Additional file 1:** **Table 5. **Residual correlations.

**Additional file 2: Table 6. **Ordinal sum score to interval score transformation table.

**Additional file 3: Table 7. **Item location parameters.

## Data Availability

The datasets generated and/or analyzed during the current study are available in the Open Science Framework repository: https://doi.org/10.17605/OSF.IO/TXVW2.
