# Supplementary material for: A psychometric evaluation of the Swedish translation of the Perceived Stress Scale: a Rasch analysis
Source: BMC Psychiatry. 2023 Sep 22;23:690. doi: 10.1186/s12888-023-05162-4 (PMC10515233; doi:10.1186/s12888-023-05162-4)
Supplement: Supplementary file 1 — Additional file 1: Table 5. Residual correlations. [file 12888_2023_5162_MOESM1_ESM.docx]

**Supplementary material**

| **Table 5**  *Residual correlations* | | | | | | | | | | | | | | |
| --- | --- | --- | --- | --- | --- | --- | --- | --- | --- | --- | --- | --- | --- | --- |
|  | **q1** | **q2** | **q3** | **q4** | **q5** | **q6** | **q7** | **q8** | **q9** | **q10** | **q11** | **q12** | **q13** | **q14** |
| **q1** |  |  |  |  |  |  |  |  |  |  |  |  |  |  |
| **q2** | **0.53** |  |  |  |  |  |  |  |  |  |  |  |  |  |
| **q3** | **0.4** | **0.52** |  |  |  |  |  |  |  |  |  |  |  |  |
| **q4** | -0.33 | -0.43 | -0.35 |  |  |  |  |  |  |  |  |  |  |  |
| **q5** | -0.37 | -0.52 | -0.44 | **0.58** |  |  |  |  |  |  |  |  |  |  |
| **q6** | -0.32 | -0.54 | -0.49 | **0.5** | **0.64** |  |  |  |  |  |  |  |  |  |
| **q7** | -0.4 | -0.52 | -0.44 | **0.39** | **0.46** | **0.51** |  |  |  |  |  |  |  |  |
| **q8** | **0.21** | **0.42** | **0.42** | -0.28 | -0.38 | -0.39 | -0.39 |  |  |  |  |  |  |  |
| **q9** | -0.47 | -0.46 | -0.39 | **0.42** | **0.42** | **0.38** | **0.39** | -0.32 |  |  |  |  |  |  |
| **q10** | -0.38 | -0.58 | -0.52 | **0.39** | **0.48** | **0.5** | **0.53** | -0.49 | **0.42** |  |  |  |  |  |
| **q11** | **0.42** | **0.35** | **0.3** | -0.29 | -0.35 | -0.29 | -0.34 | 0.14 | -0.47 | -0.27 |  |  |  |  |
| **q12** | 0.14 | **0.27** | **0.36** | -0.13 | -0.18 | -0.23 | -0.17 | **0.35** | -0.19 | -0.29 | 0.06 |  |  |  |
| **q13** | -0.21 | -0.45 | -0.36 | **0.27** | **0.35** | **0.35** | **0.31** | -0.46 | **0.23** | **0.49** | -0.17 | -0.3 |  |  |
| **q14** | **0.31** | **0.54** | **0.51** | -0.43 | -0.51 | -0.57 | -0.5 | **0.47** | -0.37 | -0.6 | **0.25** | **0.26** | -0.5 |  |

Note. *Relative cut-off value (highlighted in bold) is 0.172, which is 0.2 above the average correlation of all item-pairs.*
