# Supplementary material for: A psychometric evaluation of the Swedish translation of the Perceived Stress Scale: a Rasch analysis
Source: BMC Psychiatry. 2023 Sep 22;23:690. doi: 10.1186/s12888-023-05162-4 (PMC10515233; doi:10.1186/s12888-023-05162-4)
Supplement: Supplementary file 2 — Additional file 2: Table 6. Ordinal sum score to interval score transformation table. [file 12888_2023_5162_MOESM2_ESM.docx]

| **Table 6**  *Ordinal sum score to interval score transformation table* | | |
| --- | --- | --- |
| **Ordinal sum score** | **Logit score** | **Logit std.error** |
| 0 | -4.000 | 1.295 |
| 1 | -3.167 | 0.935 |
| 2 | -2.513 | 0.755 |
| 3 | -2.037 | 0.662 |
| 4 | -1.651 | 0.604 |
| 5 | -1.320 | 0.563 |
| 6 | -1.026 | 0.532 |
| 7 | -0.760 | 0.508 |
| 8 | -0.516 | 0.489 |
| 9 | -0.288 | 0.473 |
| 10 | -0.073 | 0.460 |
| 11 | 0.130 | 0.450 |
| 12 | 0.325 | 0.441 |
| 13 | 0.513 | 0.434 |
| 14 | 0.696 | 0.430 |
| 15 | 0.875 | 0.427 |
| 16 | 1.051 | 0.426 |
| 17 | 1.227 | 0.427 |
| 18 | 1.405 | 0.430 |
| 19 | 1.586 | 0.437 |
| 20 | 1.773 | 0.446 |
| 21 | 1.970 | 0.460 |
| 22 | 2.182 | 0.479 |
| 23 | 2.414 | 0.506 |
| 24 | 2.678 | 0.544 |
| 25 | 2.990 | 0.601 |
| 26 | 3.385 | 0.692 |
| 27 | 3.952 | 0.870 |
| 28 | 4.000 | 0.888 |
