# Supplementary material for: A psychometric evaluation of the Swedish translation of the Perceived Stress Scale: a Rasch analysis
Source: BMC Psychiatry. 2023 Sep 22;23:690. doi: 10.1186/s12888-023-05162-4 (PMC10515233; doi:10.1186/s12888-023-05162-4)
Supplement: Supplementary file 3 — Additional file 3: Table 7. Item location parameters. [file 12888_2023_5162_MOESM3_ESM.docx]

| **Table 7**  *Item location parameters* | | | | | |
| --- | --- | --- | --- | --- | --- |
| **Item** | **Threshold 1** | **Threshold 2** | **Threshold 3** | **Threshold 4** | **Average location** |
| **q1** | -0.43 | 0.87 | 2.21 | 3.17 | 1.46 |
| **q2** | -0.54 | 0.67 | 1.43 | 2.62 | 1.05 |
| **q3** | -2.02 | -0.63 | 0.83 | 1.69 | -0.03 |
| **q8** | -1.68 | -0.16 | 1.17 | 1.87 | 0.3 |
| **q11** | -0.68 | 0.89 | 1.79 | 2.94 | 1.23 |
| **q12** | -2.95 | -1.67 | -0.36 | 0.85 | -1.03 |
| **q14** | -0.51 | 0.76 | 1.75 | 2.22 | 1.06 |
